# Acute Orbital Apex Syndrome Caused by Idiopathic Sclerosing Orbital Inflammation

**DOI:** 10.3390/diagnostics12123003

**Published:** 2022-12-01

**Authors:** Chung-Chih Chang, Yung-Ching Chang, Kuei-Ying Su, Yuan-Chieh Lee, Fang-Ling Chang, Ming-Hsun Li, Yen-Chang Chen, Nancy Chen

**Affiliations:** 1 School of Medicine, Tzu Chi University, Hualien 970, Taiwan; 2 Departments of Ophthalmology, Hualien Tzu Chi Hospital, Buddhist Tzu Chi Medical Foundation, Hualien 970, Taiwan; 3 Division of Allergy, Immunology and Rheumatology, Hualien Tzu Chi Hospital, Buddhist Tzu Chi Medical Foundation, Hualien 970, Taiwan; 4 Department of Ophthalmology and Visual Science, Tzu Chi University, Hualien 970, Taiwan; 5 Institute of Medical Sciences, Tzu Chi University, Hualien 970, Taiwan; 6 Department of Anatomical Pathology, Hualien Tzu Chi Hospital, Buddhist Tzu Chi Medical Foundation, Hualien 970, Taiwan; 7 Department of Pathology, School of Medicine, Tzu Chi University, Hualien 970, Taiwan

**Keywords:** idiopathic sclerosing orbital inflammation, orbital apex syndrome, ophthalmoplegia, vision loss

## Abstract

Idiopathic sclerosing orbital inflammation (ISOI) is a distinct entity among other orbital diseases. It is characterized by marked fibrosis and inflammatory cell infiltration that can damage orbital structures. Clinical manifestations were variable, including ocular and periocular redness, proptosis, and pain. Ocular motor restrictions and optic nerve dysfunction might occur in severe cases. We herein report a patient of ISOI who presented with total ophthalmoplegia and acute vision loss. His symptoms were relieved mainly as his vision improved to 20/25 after receiving corticosteroid and immunosuppressant therapies. Therefore, ISOI should be one of the deferential diagnoses when we encounter cases with acute orbital apex syndrome. With prompt evaluation and in-time treatment, a favorable outcome is possible.

Idiopathic orbital inflammation (IOI), previously known as pseudotumor, is one of the possible etiologies other than infections and neoplasms when orbital symptoms are encountered. Idiopathic sclerosing orbital inflammation (ISOI), a pathological subgroup of IOI, is characterized by marked fibrosis and inflammatory cell infiltration that can damage orbital structure and vision by mass effects [1,2]. ISOI is more insidious and has more chronic disease progression than IOI, and therefore is more challenging to recognize and treat [3]. We had a 68-year-old patient referred to our emergency department due to abrupt ophthalmoplegia and vision loss in his left eye. His visual acuity was HM 50 cm OS. His left eye had a relative afferent pupil defect and ophthalmoplegia in all directions. The fundoscopy of the left eye showed a prominent swollen disc, retinal venous dilatation, and chorioretinal folds (Figure 1A). Coronal T1W1 magnetic resonance imaging (MRI) of orbit demonstrated an inflammatory mass filling the left retrobulbar region with encasement of the optic nerve (Figure 1B). Axial T1W1 MRI of the orbit showed a diffuse lesion with homogenous contract enhancement infiltrating intraconal space through extraocular muscles and retrobulbar region and extending to the cavernous sinus (Figure 1C). 

An orbitotomy for orbital tissue biopsy was performed. Intraoperatively, the sclerosing fragments were taken piecemeal, and the histopathological study revealed granulomatous inflammation composed of epithelioid cells, multinucleated giant cells, lymphoplasmacytic infiltration, and fibroblasts with marked fibrosis (Figure 2A). The multinucleated giant cells are CD68-positive inflammatory foreign body type multinucleated giant cells (Figure 2C,D). No foamy histiocyte (xanthoma cell) or Touton Giant cell is seen.

Another tissue section presented lymphoplasmacytic infiltration with significant fibrinoid necrosis in the wall of vessels, which suggested vasculitis (Figure 2B). No fungus is seen by periodic Acid-Schiff (PAS) and Grocott Methenamine Silver (GMS) Stain. The acid-fast stain is negative for Mycobacterium.

Given that his serum IgG4 was in a normal range, the IgG4-bearing plasma cell per HPF and IgG4/total IgG ratio were low, and c-ANCA/p-ANCA tested negative, the provisional diagnosis was ISOI. Although Erdheim Chester disease and adult xanthogranuloma were among the differential diagnosis, the clinical presentation was not likely. After being given methylprednisolone 250 mg qw for four weeks and methotrexate 12.5 mg once a week for three months while gradually tapping prednisolone to 5 mg qd, the ophthalmoplegia was significantly relieved, while the visual acuity in the left eye improved to 20/50. The orbital MRI revealed a remarkable inflammation resolution. However, the sclerosing mass remained large (Figure 3). Fortunately, the disc edema resolved, and his visual acuity improved to 20/25 eleven months after the initial presentation.

Since the epic research of ISOI by Rootman et al. [2], it has been more than two decades of paradigm evolution for this insidiously progressive subgroup of IOI. It was previously recognized as orbital lipo-granuloma due to fat necrosis, resulting in granulomata, fibrosis, and degenerative changes [4]. The clinical features of the ISOI resulted more from infiltration and mass effect than from inflammation [2]; therefore, the most common manifestations were pain, proptosis, and ocular motility restrictions [5]. Its histopathology is characterized by the destruction of the peri-orbital fat and dense collagen deposition with infiltration by inflammatory cells [2]. The differential diagnosis of such orbital lesions includes primary and secondary neoplasm, orbital inflammatory diseases such as granulomatosis polyangiitis, thyroid orbitopathy, sarcoidosis, tuberculosis, and immunoglobulin G4-related ophthalmic disease (IgG4-ROD). The previous studies aimed to cull ISOI from IOI, for the ISOI represented a unique entity rather than a continuum of IOI change [2]. Moreover, increasing evidence found that some previously presumed ISOI were IgG4-ROD [6]. These findings showed that the IgG4-unrelated ISOI had distinct clinicopathological features, including unilaterality and the orbit lesions without the lacrimal gland involvements [6,7].

The literature review of Zborowska et al. [8] showed that more than half of IOI with extra orbital extensions manifested as reduced visual acuity, afferent pupillary defect, ophthalmoplegia, and disc change. The study also suggested that such cases were associated with sclerosing type and 23% were left permanently disabled, which was most commonly unilateral blindness or other neurologic deficits. In another study of ISOI, there were 13 patients (42%) with orbital apex involvement [1]. Reduced visual acuity of the affected eye was recorded in 12 of 30 ISOI patients, and their visual acuity remained unchanged (5/12) or worse (5/12) after treatment. Four patients with visual acuity worse than 20/80 all had orbital apex lesions [1]. Therefore we speculated that ISOI is prone to cause orbital apex syndrome (OAS), characterized by simultaneous vision loss and ophthalmoplegia, as in our patient. OAS might result from the site of the inflammation and the sclerosing histological features. In our patient, the tumefactive lesion occupied the whole retrobulbar space and extended beyond the orbit, affecting the pterygopalatine fossa and the cavernous sinus. Its mass effect caused optic nerve compression, choroidal folds, and total ophthalmoplegia.

Previous reviews and case series of ISOI concluded that its treatment was nonspecific and the response was variable. Due to the nature of irreversible fibrosis, early diagnosis, younger onset, and prompt initiation of therapy were regarded as factors of better prognosis [3,9]. A retrospective case series compared the treatment outcomes of ISOI cases to that of IgG4-ROD patients, and revealed that although there was no significant difference in the response to first-line steroid treatments in both groups; however, the clinical sequelae were different. Among the 22 ISOI patients, 10 achieved medication-free remission, leaving one with vision loss and four with ocular motility dysfunctions. On the contrary, none had functional defects in the IgG4-ROD patients’ group, indicating a more aggressive course of ISOI cases than the IgG4-ROD ones [7]. In a review of 61 ISOI patients, the combination therapy group (83.9%) had a better response than the steroid-alone group (66.6%). The combination therapy included steroids, radiotherapy, immunosuppressants, cytotoxic agents, biologic agents, or their combinations [5]. There were anecdotal successes with treatments such as Rituximab and mycophenolate mofetil in steroid-resistant ISOI cases [10,11,12]. Researchers proposed that a standardized clinical grading scale and multidisciplinary cooperation are needed to achieve consensus on optimal treatment regimens [5].

In our study, the ISOI involved the orbital apex and extended beyond orbit. It presented as abrupt unilateral total ophthalmoplegia and vision loss. Combining orbital imaging, pathological studies, and a thorough rheumatology survey, the diagnosis of ISOI was made prudently. The corticosteroids and immunosuppressant therapies were administrated for five months, and the patient achieved good visual and functional outcomes at the eleventh-month follow-up. The course demonstrated that both inflammation and mass effect contributed to the orbital apex syndrome of ISOI, and the response could be favorable if managed promptly.

## Figures and Tables

**Figure 1:**
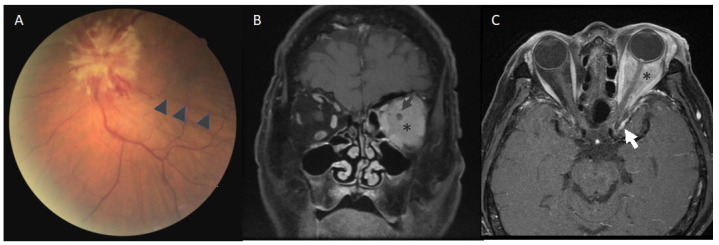
(**A**) Fundoscopy of the left eye showed a prominent swollen disc, retinal venous dilatation, and chorioretinal folds (arrowhead). (**B**) Coronal magnetic resonance imaging (MRI) (T1W1) demonstrated the left retrobulbar mass (aristek) encasing the optic nerve (arrow). (**C**) Axial MRI (T1W1) showed a homogenous lesion (aristek) extending from the left peribulbar space to the cavernous sinus (arrow).

**Figure 2:**
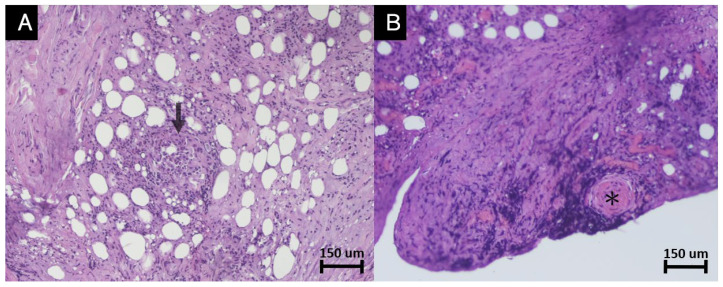

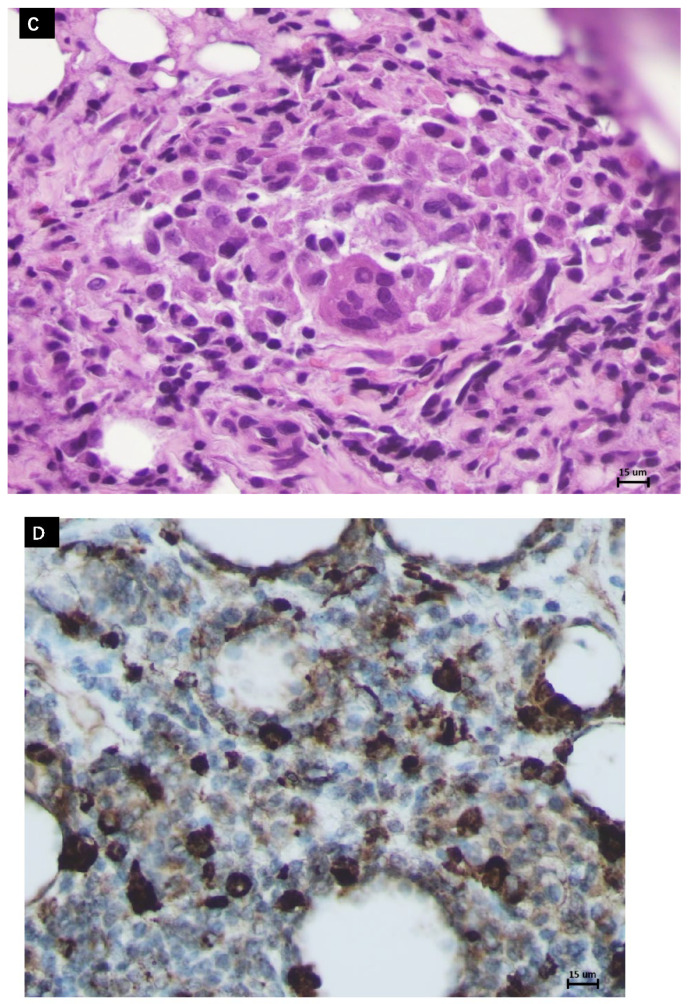
The histopathological study revealed a lymphoplasmacytic and fibroblastic infiltration with marked fibrosis. (**A**) The multinucleated giant cells (arrows) indicated prominent granulomatous inflammation (H and E stain, origin X100). (**B**) Lymphoplasmacytic infiltration with significant fibrinoid necrosis in the wall of vessels (asterisk) suggested vasculitis (H and E stain, origin X100). (**C**) Higher power field to highlight the inflammatory infiltrate. (**D**) The multinucleated giant cells are CD68-positive inflammatory foreign body type multinucleated giant cells.

**Figure 3:**
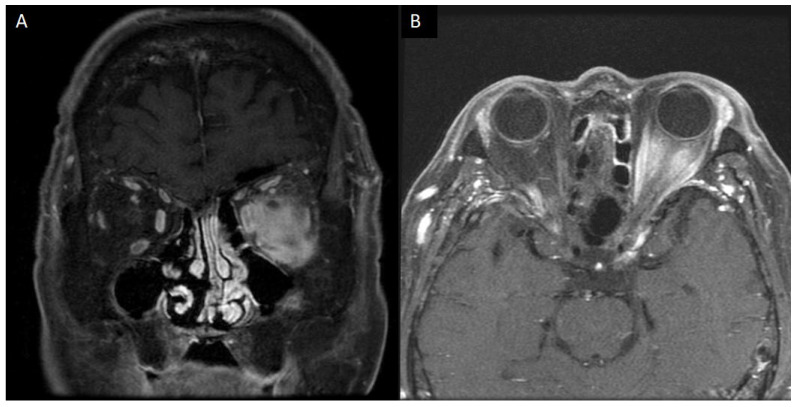
After treatment, the orbital MRI revealed a remarkable resolution of inflammation (**A**,**B**).

## Data Availability

Not applicable.
